# Learning to Care for the Older People: An Urgent Need in the Daily Practice of Oncologists

**DOI:** 10.3390/jcm11113149

**Published:** 2022-06-01

**Authors:** Gerardo Rosati

**Affiliations:** Medical Oncology Unit, “San Carlo” Hospital, 85100 Potenza, Italy; oncogerry@yahoo.it; Tel.: +39-0971-612273; Fax: +39-0971-613000

Cancer is the most widespread and prominent health issue worldwide and its incidence is only exceeded by cardiovascular diseases [1]. While cancer diagnosis and treatment have undoubtedly been delayed and curtailed in the past two years by the aftermath of the coronavirus (COVID-19) pandemic, it is likely that returning to normal lifestyle habits will lead to an increased incidence of cancer with a more advanced stage and with increased mortality [2].

More prolonged exposure to carcinogens over time and the natural impairment of the immune system over the years may explain how cancer is particularly common at geriatric age [3]. The number of individuals aged ≥70 years diagnosed with cancer was approximately 7.2 million (37% of all cases) worldwide in 2020 and is expected to almost double by 2040 [4]. The socio-economic development of Western countries and the consequential increase in life expectancy lead to a close correlation between cancer and older patients [5,6]. This has significant implications for the organization of national health services, and increased use of economic resources will be required over the next few years [7].

Age alone cannot limit access to cancer treatment [8]. In some countries, such as Australia, this would even be considered discriminatory [9]. First of all, the first mistake that every oncologist should avoid making is not considering the chronological age of the geriatric patient in the decision-making approach, but instead focusing on his/her biological age [10]. Even so, physicians face many difficulties when treating an older patient for two essential reasons: (i) the under-representation of these individuals in randomized clinical trials [11], and (ii) the extreme heterogeneity of older people who require customized interventions, different from standard interventions [12]. As a result, there is no evidence-based approach for these patients, and a lack of knowledge of such shortcomings could lead to under- or over-treatment, prejudicing the benefits of potentially effective therapy. In addition, aging involves changes in health, but also in functional, cognitive, emotional, nutritional, and social status; it affects cancer treatment due to shortened life expectancy, different tolerability of therapies, changing patient expectations, and sometimes even the inability to care for the patient due to social barriers and the lack of a caregiver [13].

In order to correctly frame each patient, allowing, on the one hand, the most appropriate therapy and prognostic judgment and, on the other hand, avoiding unacceptable toxicities, guidelines have been defined that make it necessary to implement a geriatric assessment (CGA) in the management of older cancer patients [14,15]. However, completing a CGA requires not only numerous resources, but also expertise in its management and data interpretation, the collaboration of other professional figures such as geriatricians and nurses, and it takes a lot of time away (estimated between 30 and 60 min) from the clinical activity of oncologists [16,17,18]. Consequently, new, accurate tools such as the Geriatric-8 (G8) and the Vulnerable Elders Survey-13 (VES-13) have been identified as rapid screening tools due to their sensitivity and specificity, and they ensure that a CGA is only administered to patients with actual impairments in CGA domains and who are likely to benefit from geriatric intervention [19]. The integration of these instruments will identify fit older patients who will be able to benefit from treatments that can also be proposed to younger patients, frail patients who can be assigned to the best supportive care, and patients who can be categorized as vulnerable and assigned to less intensive therapies that can be adapted on a case-by-case basis [7].

In recent years, many researchers have, thus, defined recommendations and guidelines that highlight some key issues in the management of older patients who undergo surgery [20], as well as in the treatment of advanced diseases, both for cancers typical of this age, such as those of the prostate [21], and for those with more widespread incidence and mortality, such as those of the lung [22,23], colon [7], gastric [24], and breast [25].

Nevertheless, with the new introduction of immunotherapy, monoclonal antibodies, cyclin inhibitors, and targeted therapies in the therapeutic armamentarium, oncologists now face difficult choices in caring for their older patients, not only due to the lack of knowledge in this setting of individuals, but also due to the need to outweigh their reduced life expectancy and the costs of innovative drugs. The articles contained in the Special Issue “Clinical Frontiers in Geriatric Oncology” of the *Journal of Clinical Medicine* take stock of these new therapies in the treatment of major cancers and verify their impact and sustainability at geriatric age.

Overall, I believe that the key points raised in this editorial might help oncologists to interpret the findings of the treatment of older patients in their daily clinical practice.
